# Pulp Responsiveness of Healthy Non-Pathological Teeth Following Surgical Enucleation of Cysts

**DOI:** 10.3390/dj13030116

**Published:** 2025-03-05

**Authors:** Syed Nabil, Muhd Fazlynizam Rashdi, Abd Jabar Nazimi

**Affiliations:** 1 Department of Oral and Maxillofacial Surgery, Faculty of Dentistry, Universiti Kebangsaan Malaysia, Kuala Lumpur 50300, Malaysia; mohdnazimi@ukm.edu.my; 2 Department of Oral and Maxillofacial Surgery, Hospital Canselor Tuanku Muhriz, Universiti Kebangsaan Malaysia, Kuala Lumpur 56000, Malaysia; fazlynizam@hctm.ukm.edu.my

**Keywords:** tooth vitality, sensibility test, jaw cysts, enucleation, electric pulp testing

## Abstract

**Background/Objectives**: Odontogenic cysts are pathological cavities lined by cells arising from odontogenic epithelial cells, occurring mostly on the tooth-bearing areas of the jaws. It is common to find that the apices of the teeth around the cyst are within the cyst’s cavities due to its expansion. This study aims to assess the outcome of cyst enucleation on the associated teeth, specifically the latter’s responsiveness after cyst enucleation. **Methods**: This retrospective study examined a sample of patients who had been previously treated for odontogenic cysts from 1 January 2000 to 31 December 2021. A list of patients was obtained and included whether they met the imposed inclusion criteria. The data collected included the patients’ preoperative and postoperative electric pulp testing readings and their timings. **Results**: In total, 77 individual teeth from 19 patients were included after meeting the inclusion/exclusion criteria. Overall, 57 out of the 77 (74%) teeth were responsive following long-term follow-up. Among the 57 teeth with a positive response, 8 teeth were initially non-responsive and regained their responsiveness after a period of time. Pulp responsiveness recovery was seen even 300 days after surgery. **Conclusions**: It is not certain that a tooth with apices involved in a cyst cavity will be non-vital following enucleation. It is recommended that these teeth be reassessed for a minimum of 10 months postoperatively before proceeding with root canal treatment.

## 1. Introduction

Odontogenic cysts are pathological epithelial-lined cavities containing fluid or semi-fluid that arise from the epithelial remnants of teeth, such as reduced enamel epithelium (REE), rests of Malassez, and rests of Serres. According to the fifth edition (2022) of the World Health Organization (WHO) Classification of Head and Neck Tumors, the umbrella term “cyst of the jaws”, also known as odontogenic cysts, has been used generally to describe any cystic lesion involving the maxilla and the mandible. These cysts are subdivided into three types: inflammatory odontogenic cysts, developmental odontogenic cysts, and other cysts of the jaws [1]. Inflammatory odontogenic cysts include radicular cysts and inflammatory collateral cysts, whereas dentigerous cysts, gingival cysts, and odontogenic keratocysts (OKCs) are categorized as developmental odontogenic cysts. A radicular cyst is the most common type of jaw cyst, followed by a dentigerous cyst [2]. A radicular cyst develops as a sequela of the inflammation of the pulp and periodontium that is usually associated with a non-vital tooth. On the other hand, a dentigerous cyst, also known as a follicular cyst, results from the accumulation of fluid between the REE and the crown of an unerupted tooth. For both lesions, patients are usually asymptomatic, and these are often found incidentally through radiographic examination.

Various methods can be utilized to assess the pulpal status. Although commonly used interchangeably, pulp vitality is defined as the presence of blood flow in the pulp, while pulp sensibility or responsiveness is defined as the pulp’s ability to produce a response to a stimulus [3]. Directly inspecting the pulp histological section is the gold standard for determining vitality status, but it is not practical in most situations. Indirect methods such as pulp responsiveness tests, including thermal tests and the electric pulp test (EPT), are more commonly used to indicate pulpal health. These responsiveness tests, however, only assess the neural response and do not consider the vascular circulation that may lead to false-positive or false-negative responses [3,4]. Tooth vitality tests, therefore, are a better measure of pulpal health than responsiveness tests, but tooth responsiveness tests are more commonly used due to their practicality.

The two common treatments for jaw cysts are total enucleation or the less-invasive marsupialization. The enucleation of cysts, in particular, has been proven to be a predictable method of treatment, consisting of the complete removal of the cystic capsule. When a jaw cystic lesion is of a significant size, the teeth in that part of the jaw may be displaced, or their roots may be included in the cystic cavity. The vitality of the adjacent non-pathologic teeth, with their apices included within the cyst, is of unknown prognosis. Clinicians often need to decide whether prophylactic root canal treatment (RCT) of the adjacent non-pathologic teeth is necessary, since devitalization of these teeth is considered unavoidable while enucleating the cystic membrane [5]. This study, therefore, aimed to provide insight into the effect of cyst enucleation on the responsiveness of the adjacent teeth involved in cystic jaw lesions and their projected prognosis over time.

## 2. Materials and Methods

This retrospective study examined a sample from the population of patients who were previously treated for odontogenic cysts at the Oral and Maxillofacial Surgery Department at Hospital Canselor Tuanku Muhriz, Universiti Kebangsaan Malaysia (HCTM UKM), from 1 January 2000 to 31 December 2021. This study obtained ethical approval from the UKM ethical committee, under reference number JEP-2021-817. A list of patients treated for odontogenic cysts was obtained from the department’s operating theater list and outpatient clinic appointment census. From these lists, patients were screened according to the study’s inclusion criteria.

The following inclusion criteria were applied: (1) patients who underwent a cyst enucleation procedure in HCTM UKM; (2) lesions must have been histopathologically proven to be only radicular and/or dentigerous cysts; (3) the “associated tooth” must have had responsiveness testing via EPT conducted preoperatively and postoperatively; (4) the “associated tooth” must have had a positive response preoperatively; and (5) a preoperative radiograph of the cysts must have been available for the assessment of root involvement with the cyst. The exclusion criteria, meanwhile, were as follows: (1) patients below the age of 12 years old; (2) the associated tooth was non-responsive preoperatively or had not been assessed for preoperative responsiveness via EPT preoperatively; and (3) the tooth had other possible clinical causes of non-responsiveness, such as having a large filling in proximity to the pulp chamber or resorption of the roots. Once subjects meeting the criteria were identified, the relevant information was collected from their medical records. The information collected was demographic data (age, gender, and race) and clinical data (comorbidities, diagnosis, pathogenic tooth, associated tooth, and preoperative and postoperative responsiveness test readings). The preoperative radiographs were reviewed to assess the tooth that was involved with the cyst. For this study, the “associated tooth” was defined as any tooth with its apices within the cyst cavity in the preoperative radiograph that was not extracted during surgery.

The main outcome was the result of the responsiveness test of the associated tooth (pre- and postoperative responsiveness test value). All demographic and clinical factors were explored for any association with the responsiveness test outcome. The responsiveness test values recorded were statistical tests performed using the Statistical Package for Social Science (SPSS version 26.0). Numerical data were presented as the mean, standard deviation, and range; *p* < 0.05 was taken to indicate statistical significance.

## 3. Results

We identified 88 patients who underwent cyst enucleation surgery following screening of the operative records of all patients who had undergone surgery under general anesthesia within the study period. In 25 of the 88 cases, medical records were not available, and thus, 63 cases were assessed for eligibility based on the pre-determined inclusion and exclusion criteria. Of these 63 patients, there were 32 males and 31 females with a mean age of 44 years (range 13–79 years). The majority of the patients were Malay (57.1%), followed by Chinese (27.0%), Indian (6.3%), and other ethnicity (9.6%). Following the inclusion/exclusion criteria assessment, 44 patients were excluded. Among these, 12 were excluded due to diagnoses that were not radicular cysts or dentigerous cysts. These other diagnoses included but were not limited to odontogenic keratocysts and unicystic ameloblastoma. Another 30 patients were excluded for not having either preoperative or postoperative EPT readings. Another two patients were excluded due to the unavailability of preoperative radiographs. Thus, there were a total of 19 patients who satisfied all the inclusion criteria. The majority of them had radicular cysts (79%), while the remainder had dentigerous cysts (11%). Cases with dentigerous cysts involved more “associated teeth”, with 75% of cases involving five or more teeth, while only 33% of cases of radicular cysts had similar involvement. There was a significant difference in the ethnic representation between the two types of cysts included in this study. The details of the 19 patients are described in Table 1.

**Figure 1:**
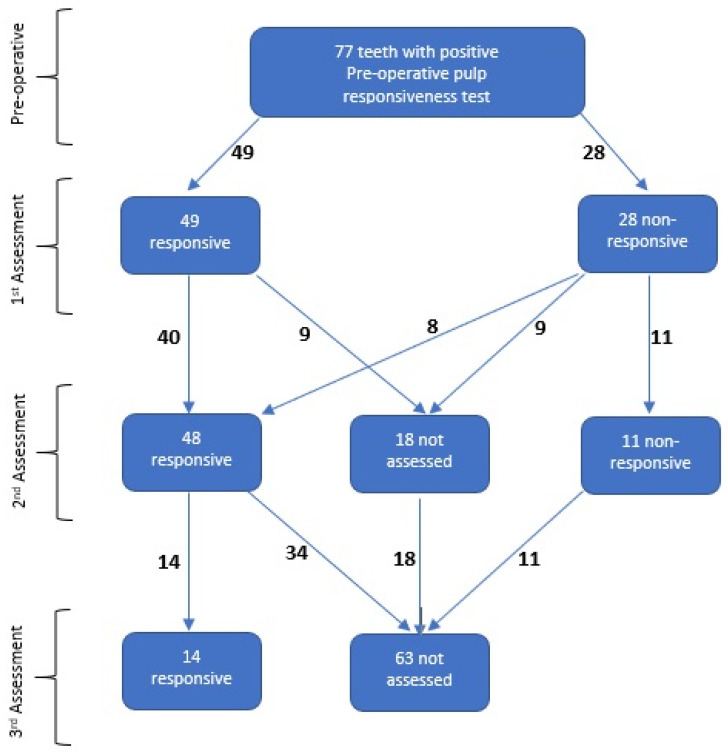
Flow of the assessment of the responsiveness of teeth.

From the 19 included patients, there were 77 teeth that were considered to be “associated teeth” that underwent EPT testing pre- and postoperatively. A total of 77 preoperative and 150 postoperative responsiveness tests were recorded. Among the 150 test readings, 77 teeth had at least one postoperative responsiveness assessment, another 59 had a second responsiveness assessment, and 14 teeth had a third assessment (Figure 1). Out of the 77 teeth, 28 (36%) were not responsive during the first postoperative assessment. Among the teeth which underwent a second responsiveness assessment, the percentage of non-responsive teeth dropped to 14% (11 out of 59). None of the tested teeth had negative EPT readings on the third assessment (Table 2). Comparing radicular and dentigerous cysts, during the first reading, both had almost similar percentages of non-responsive teeth, with 35% vs. 39%. However, during the second reading, most of the dentigerous cysts regained their responsiveness, with responsive readings seen in 19% versus 4% for radicular and dentigerous cysts, respectively. When relating the period of time with the responsiveness assessment, the majority of the first reading (55%) was performed within 60 days after surgery. Another 35% had their first assessment between day 61 and 150 postoperatively. Only 9% of teeth were assessed after 150 days. For the second reading, 44% of the teeth were assessed after day 300. All third assessments were conducted after 300 days post surgery (Table 3).

When assessing the relationship between the time of the assessment and the responsiveness outcome, there was a progressive decrease in the percentage of non-responsive teeth over time. When the teeth were assessed less than 60 days following surgery, 44% were non-responsive. This decreased to only 10% of teeth being non-responsive at assessment after 300 days following surgery. There was a significant relationship between the responsiveness outcome and the timing of the assessment (Table 4). Among the 28 non-responsive teeth in the first reading, only 19 were tested a second time, among which 8 regained responsiveness, while the remaining 11 were still negative. This meant a 42% (8 out of 19) recovery rate. Most responsiveness recovery was detected after day 150 post surgery (87.5%). Only one (12.5%) recovery was detected before 150 days post surgery (Table 5). All eleven teeth that were non-responsive during the second postoperative assessment did not undergo a third assessment either because they had been lost to follow-up or were referred for RCT.

The eight teeth that recovered their responsiveness were related to six patients (Table 6). All patients were female, with ages ranging from 25 to 49 years old. Two patients had two teeth that recovered, while the others had only one. Five out of the six cases were diagnosed as radicular cysts and located at the anterior jaw, while the single dentigerous cyst case originated at the posterior jaw. All teeth that had their responsiveness recovered were maxillary teeth.

## 4. Discussion

There is an established inter-relation between a tooth’s non-responsiveness to the responsiveness test and the state of total necrosis [6]. EPT, as one of the responsiveness tests, is a tool that measures the responsiveness of tooth pulp by relying on subjective assessment by eliciting a response from the patient [7]. EPT works by producing enough electrical energy through the enamel, past the dentine, and into the pulp to elicit a measurable pulpal response. As the test is conducted using electrical current transmittance, it is possible to produce a false-positive result due to failure to establish adequate isolation or due to heavy restorations, both of which are possible causes of an electrical current being transmitted to the adjacent tooth [8]. Compared to cold tests, EPT is found to be inferior in assessing pulp responsiveness [6]. Even though vitality tests such as pulse oximetry and laser Doppler flowmetry are more accurate in assessing pulpal health, these methods are less accessible, expensive, and technically difficult to use. Hence, pulp responsiveness tests such as EPT still provide valuable diagnostic information on pulpal health in daily practice, provided that the clinician uses correct and appropriate techniques [8].

When a tooth is identified to be involved in a cystic lesion, the pathological causal tooth will usually undergo elective RCT. RCT is also further indicated when the adjacent non-pathological tooth is also not vital preoperatively. The controversies are mostly in managing teeth with apices within the cyst that are still vital preoperatively, which are called “associated teeth” in this study [9]. In our center, we are usually faced with such decisions when treating dentigerous cysts and radicular cysts but not odontogenic keratocysts, wherein the associated teeth are extracted during enucleation surgery to prevent recurrence. This is the reason for only accepting dentigerous and radicular cysts in this study. In our center, RCT are usually performed preoperatively only on pathological teeth and non-responsive associated teeth. This approach, however, necessitates regular and extended follow-up after surgery to assess the responsiveness and need for intervention. Teeth have been postulated to be responsive even after cyst enucleation due to a few possible reasons [10]. First, the presence of stable blood clots in the contained cavity post-enucleation could allow neurogenesis and angiogenesis. It is also possible that the vital pulp with a pulp stem allows a high potential for recovery; in addition, collateral reinnervation to the affected pulp can occur, especially if there are teeth with collateral canals not within the cystic cavity.

The recovery of responsiveness is not a new concept. This is a situation that has been reported mostly in teeth that initially lost their responsiveness due to trauma or even autotransplantation cases [11,12]. The recovery of responsiveness among teeth with apices within the cyst cavity, however, is less discussed. It is accepted that the responsiveness of teeth might be preserved with less-invasive surgery, such as marsupialization [13]. However, even for enucleation, there have been some reported cases of teeth responsive to the responsiveness test after surgery. Pittl et al. reported recovery of the responsiveness of associated teeth in the case of a large OKC at the lower mandible, even after enucleation and application of Carnoy’s solution [14]. Another reported case had two anterior teeth regain responsiveness to the vitality test 1 year post surgery and remain vital 4 years later [15]. More recently, three teeth that were associated with a radicular cyst recovered their responsiveness 1 year after surgery [10]. These individual case report findings are corroborated by a recently published case series that followed up on the vitality of associated teeth for three years after cyst enucleation surgery [16]. They found that, after 3 years, only 19 of 103 associated teeth were non-responsive. This means that 82% of the associated teeth avoided unnecessary RCT [16]. Our study had 49 teeth that were responsive and another 8 that recovered responsiveness. This makes a total of 57 out of the 77 (74%) that were responsive following surgery, which is lower than the findings from Niu et al. This finding can be due to the fact that the teeth that were non-vital during the first or second test would have been sent for RCT earlier than the observed time of 3 years in that study.

Time is an important aspect of responsiveness recovery. Niu et al. allowed 3 years to elapse for responsiveness recovery [16]. They found that, upon assessment after 24 months, the responsiveness recovery of a further eight teeth was found compared to the assessment at 12 months. No further tooth had a responsiveness recovery at 36 months. The other reported cases also had recovery only after a lengthy period of time, finding a recovery of responsiveness only after 1 year of follow-up [10,15]. Based on our results, we found a significant relationship between responsiveness recovery and time and that responsiveness recovery could be seen even 300 days after surgery. Our findings, supported by previous studies, suggest that the recovery of responsiveness can take time; however, there is a cutoff period where no further recovery can be seen. This cutoff period, however, is still not clear, although 1 year seems appropriate.

Some interesting findings from this study are that the eight teeth from the six patients that showed responsiveness recovery all belonged to female patients and maxillary teeth. In addition, all but one case involved radicular cysts. It is unclear how these factors relate to the capacity of a tooth to recover vitality. One possible explanation is that the potential for the recovery of vitality in the maxilla is related to the fact that it has a higher vascularity compared to the mandible [17]. Further investigations regarding the factors affecting the potential for recovery are needed to help better inform decisions on the need for elective or early RCT for teeth that lose responsiveness postoperatively.

This study is not without its limitations. Firstly, as discussed earlier, EPT does not provide a true measure of tooth pulpal health, as it only provides a sensory response to a stimulus. Furthermore, a cold test, which is said to be a more accurate test, was not conducted in our series of patients [6]. EPT results are also dependent on the patient’s subjective measure of the stimulus, which may vary from one person to another and also across different time periods. As this study was carried out by retrospectively assessing clinical records, there was no way to standardize the test or its timing. There was a high dropout rate, likely due to the nature of this study, where the test results might have influenced the clinician to stop further testing either because a tooth was already responsive, or when it was not responsive, the clinician may have sent the patient for RCT straight away. This study does, however, provide conclusive evidence that the loss of responsiveness is not a definite outcome for associated teeth following cyst enucleation. Although it is not clear which tooth has a better chance of recovering responsiveness, our study suggests that a minimum of 300 days should be allowed before deciding on the need for RCT.

## 5. Conclusions

In conclusion, this study found that there is a possibility of the recovery of the vitality of a tooth with apices within a cyst cavity. This study suggests that a tooth should be observed for a period of time before any recovery in responsiveness may be seen. The implication of this finding is that, in managing jaw cysts, elective RCT for the “associated tooth” might not be needed prior to enucleation surgery.

## Figures and Tables

**Table 1:** Demographic and clinical data for the included patients.

Variables	No. of Patients (N (%))			
	Radicular Cyst (N = 15)	Dentigerous Cyst (N = 4)	Total (N = 19)	*p*-Value
Gender:				* 1.00
Male	7 (46.7)	2 (50.0)	9 (47.4)	
Female	8 (53.3)	2 (50.0)	10 (52.6)	
Ethnicity:				* 0.02
Malay	11 (73.3)	1 (25.0)	12 (63.2)	
Chinese	3 (20.0)	0 (0)	3 (15.8)	
Indian	0 (0)	2 (50.0)	2 (10.5)	
Other	1 (6.7)	1 (25.0)	2 (10.5)	
Age During Surgery (years):				** 0.52
Mean	31.67	35.75	32.53	
Standard Deviation	10.43	13.65	10.89	
Minimum	18	23	18	
Maximum	49	49	49	
Teeth Associated with Cyst:				* 0.42
1–2 teeth	2 (13.3)	0	2 (10.5)	
3–4 teeth	8 (53.3)	1 (25.0)	9 (47.4)	
>4 teeth	5 (33.3)	3 (75.0)	8 (42.1)	

* Fisher’s exact test; ** Student’s t-test.

**Table 2:** Outcome of the responsiveness test of radicular and dentigerous cysts.

Electric Pulp Testing Times	No. of Teeth (N (%))		
	Radicular Cyst	Dentigerous Cyst	Total
Preoperative:	N = 54	N = 23	N = 77
Responsive	54 (100.0)	23 (100.0)	77 (100.0)
Not Responsive	0 (0)	0 (0)	0 (0)
Postoperative I:	N = 54	N = 23	N = 77
Responsive	35 (64.8)	14 (60.9)	49 (63.6)
Not Responsive	19 (35.2)	9 (39.1)	28 (36.4)
Postoperative II:	N = 48	N = 11	N = 59
Responsive	38 (70.4)	10 (43.5)	48 (62.3)
Not Responsive	10 (18.5)	1 (4.3)	11 (14.3)
Postoperative III:	N = 14	N = 0	N = 14
Responsive	14 (25.9)	0 (0)	14 (18.2)
Not Responsive	0 (0)	0 (0)	0 (0)

**Table 3:** Changes in the responsiveness outcome according to the number of tests.

After Surgery (Days)	Electric Pulp Testing First Reading			Electric Pulp Testing Second Reading			Electric Pulp Testing Third Reading		
	Responsive	Not Responsive	Total	Responsive	Not Responsive	Total	Responsive	Not Responsive	Total
<60	24 (49.0)	19 (67.9)	43 (55.8)	0 (0)	0 (0)	0 (0)	0 (0)	0 (0)	0 (0)
60–149	23 (46.9)	4 (14.3)	27 (35.1)	9 (18.8)	5 (45.5)	14 (23.7)	0 (0)	0 (0)	0 (0)
150–299	2 (4.1)	5 (17.9)	7 (9.1)	17 (35.4)	2 (18.1)	19 (32.2)	0 (0)	0 (0)	0 (0)
≥300	0 (0)	0 (0)	0 (0)	22 (45.8)	4 (36.4)	26 (44.1)	14 (100.0)	0 (0)	14 (100.0)
Total	49 (63.6)	28 (36.4)	77 (100.0)	48 (81.4)	11 (18.6)	59 (100.0)	14 (100.0)	0 (0)	14 (100.0)

**Table 4:** Changes in the responsiveness outcome according to the interval time after the date of surgery.

Interval Days After Date of Surgery (%)						
	<60 Days	60–149 Days	150–299 Days	≥300 Days	Total	*p*-Value
Positive reading	24 (55.8)	32 (78.1)	19 (73.1)	36 (90.0)	111 (74.0)	0.004
Negative reading	19 (44.2)	9 (21.9)	7 (26.9)	4 (10.0)	39 (26.0)	
Total	43 (100.0)	41 (100.0)	26 (100.0)	40 (100.0)	150 (100.0)	

**Table 5:** Results of the second responsiveness assessment on the 28 teeth with non-responsive results during the first postoperative assessment.

Interval After Surgery	Second Responsiveness Test Outcome (%)		
	Positive Reading	Negative Reading	Total
<60 days	0 (0.0)	0 (0.0)	0 (0.0)
60–149 days	1 (12.5)	5 (45.5)	6 (21.4)
150–299 days	3 (37.5)	2 (18.1)	5 (17.9)
≥300 days	4 (50.0)	4 (36.4)	8 (28.6)
Not assessed	-	-	9 (32.1)
Total	8 (42.1)	11 (57.9)	28 (100.0)

**Table 6:** Details on the teeth that regained responsiveness post surgery.

Patient Number	Gender	Age	Diagnosis	Location	Associated Tooth
1	Female	32	Radicular cyst	Anterior maxilla	1.1 and 2.3
2	Female	49	Radicular cyst	Anterior maxilla	1.1 and 2.2
3	Female	31	Radicular cyst	Anterior maxilla	1.1
4	Female	25	Dentigerous cyst	Right posterior maxilla	1.7
5	Female	26	Radicular cyst	Anterior maxilla	2.1
6	Female	47	Radicular cyst	Anterior maxilla	2.3

## Data Availability

The datasets generated and utilized in this study are available from the corresponding author upon reasonable request.
